# Angiopoietin-like Protein 2 Is a Multistep Regulator of Inflammatory Neovascularization in a Murine Model of Age-related Macular Degeneration*

**DOI:** 10.1074/jbc.M115.710186

**Published:** 2016-02-02

**Authors:** Manabu Hirasawa, Keiyo Takubo, Hideto Osada, Seiji Miyake, Eriko Toda, Motoyoshi Endo, Kazuo Umezawa, Kazuo Tsubota, Yuichi Oike, Yoko Ozawa

**Affiliations:** From the ‡ Laboratory of Retinal Cell Biology and; the § Department of Ophthalmology, Keio University School of Medicine, Tokyo 160-8582, Japan,; the ¶ Department of Ophthalmology, Tokyo Dental College Suidobashi Hospital, Tokyo 101-0061 Japan,; the ** Department of Molecular Genetics, Graduate School of Medical Sciences, Kumamoto University, Kumamoto, 860-8555, Japan,; the ‡‡ Department of Molecular Target Medicine Screening, Aichi Medical University School of Medicine, Aichi, 480-1195, Japan,; the §§ Core Research for Evolutional Science and Technology, Japan Agency for Medical Research and Development, Tokyo 100-0004, Japan, and; the ‖ Department of Stem Cell Biology, Research Institute, National Center for Global Health and Medicine, Tokyo 162-8655, Japan

**Keywords:** extracellular-signal-regulated kinase (ERK), inflammation, integrin, macrophage, migration, NF-kappa B (NF-KB), age-related macular degeneration, angiopoietin-like protein 2, choroidal neovascularization, inflammatory mediators

## Abstract

Choroidal neovascularization (CNV) is a pathogenic process of age-related macular degeneration, a vision-threatening disease. The retinal pigment epithelium and macrophages both influence CNV development. However, the underlying mechanisms remain obscure. Here, we focus on Angptl2 (angiopoietin-like protein 2), a cytokine involved in age-related systemic diseases. Angptl2 was originally identified as an adipocytokine and is also expressed in the eye. Using a laser-induced CNV model, we found that *Angptl2* KO mice exhibited suppressed CNV development with reduced macrophage recruitment and inflammatory mediator induction. The mediators monocyte chemotactic protein-1, interleukin-1β (*Il-1*β), *Il-6*, matrix metalloprotease-9 (*Mmp-9*), and transforming growth factor-β1 (*Tgf*-β*1*) that were up-regulated during CNV development were all suppressed in the retinal pigment epithelium-choroid of CNV models generated in the *Angptl2* KO mice. Bone marrow transplantation using wild-type and KO mice suggested that both bone marrow-derived and host-derived Angptl2 were responsible for macrophage recruitment and CNV development. Peritoneal macrophages derived from *Angptl2* KO mice expressed lower levels of the inflammatory mediators. In the wild-type peritoneal macrophages and RAW264.7 cells, Angptl2 induced the mediators via integrins α4 and β2, followed by the downstream activation of NF-κB and ERK. The activation of NF-κB and ERK by Angptl2 also promoted macrophage migration. Therefore, Angptl2 from focal tissue might trigger macrophage recruitment, and that from recruited macrophages might promote expression of inflammatory mediators including Angptl2 in an autocrine and/or paracrine fashion to facilitate CNV development. Angptl2 might therefore represent a multistep regulator of CNV pathogenesis and serve as a new therapeutic target for age-related macular degeneration.

## Introduction

Choroidal neovascularization (CNV)^2^ is the main pathogenic process associated with age-related macular degeneration (AMD), a leading cause of blindness worldwide (1). CNV disrupts Bruch's membrane and the retinal pigment epithelium (RPE), invades the subretinal space, and causes exudative changes that damage photoreceptor cells. AMD-related vision loss results in reduced social interactions and a lower quality of life in the aging population (2). Although anti-VEGF therapy, which suppresses CNV, is currently available, nonresponders to this therapy still await new therapeutic approaches (3). However, the underlying molecular mechanisms of AMD disease pathogenesis are not fully understood.

Inflammation (4, 5) and oxidative stress (5, 6) are well accepted mechanisms in the pathogenesis of CNV. Chemically mediated macrophage depletion using clodronate (7) and the absence of a macrophage recruiting factor, MCP-1 (monocyte chemotactic protein-1) (8) both suppress CNV development in mice. In contrast, mice deficient in the ATP binding cassette transporter ABCA1, which disrupts cholesterol metabolism in macrophages, enhances CNV development (9). Inflammatory mediators such as IL-1β (10, 11), IL-6 (12), MMP-9 (13), and TGF-β (14) are reported to also play roles in CNV development. Nevertheless, the whole picture of CNV pathogenesis remains to be elucidated.

Here, we focus on adipocytokine Angptl2 (angiopoietin-like protein 2), which is up-regulated with age and associated with pathogenesis of inflammatory diseases (15). Angptl2 is one of the Angptl family proteins (Angptl1–7), which possess a coiled-coil domain at the N terminus that mediates oligomerization and a fibrinogen-like domain at the C terminus (16, 17). Although angiopoietin and Angptl2 share similar protein structures, Angptl2 does not bind to the angiopoietin receptors Tie-1 and -2, suggesting that the two ligands serve different functions. Angptl2 has been linked to tumor development (15, 18), migration and metastasis of the tumor cells (19, 20), obesity (21–23), and cardiovascular disorders (24–26). Conversely, Angptl2 deficiency ameliorates inflammation in adipose tissue and subsequently improves systemic insulin resistance in diet-induced obese mice (23), and it suppresses aortic diameter enlargement in an abdominal aortic aneurysm model (27). It not only is secreted from adipose tissue (28) but also is expressed in macrophages (28) and the retina (29).

In this study, we analyzed the role of Angptl2 in CNV development using a murine model of laser-induced CNV that develops features similar to AMD (30). We also evaluated the roles of systemically circulating and locally produced Angptl2 in the pathogenesis of CNV development, as well as the effect of Angptl2 on macrophage recruitment and inflammatory mediator induction.

## Experimental Procedures

### 

#### 

##### Animals

*Angptl2* KO (*Angptl2*^−/−^) (provided by Dr. Oike, Kumamoto University, Kumamoto, Japan) and littermate WT male mice were maintained in cages with automatically controlled lighting (12 h on, 12 h off) and a stable temperature of 22 °C and fed a normal diet. The animal experiments were conducted in accordance with the Association for Research in Vision and Ophthalmology Statement for the Use of Animals in Ophthalmic and Vision Research.

##### CNV Induction

Six- to eight-week-old mice were anesthetized with 10% pentobarbital, and their pupils were dilated with 0.5% tropicamide and 0.5% phenylephrine eyedrops (Santen Pharmaceutical Co., Osaka, Japan). Laser photocoagulation was then performed at 8 spots per eye around the optic disk with a wavelength of 532 nm, a power of 200 milliwatt, a duration of 100 ms, and a spot size of 75 μm, using a slit lamp delivery system (Novus Spectra; Lumenis, Tokyo, Japan) as described previously (31).

##### Immunohistochemistry

Retinal sections (7 μm thick) fixed in 4% paraformaldehyde (32) or RPE-choroid flat mounts (12) were prepared and incubated with 0.1% Triton X-100 in PBS and 10% normal goat serum (Dako, Carpinteria, CA), as previously described. Then the samples were incubated with primary antibodies followed by Alexa Fluor secondary antibodies (Invitrogen). The primary antibodies were: anti-Angptl2 (ab35574; Abcam), anti-Rpe65 (MAB5428; Merck Millipore), anti-F4/80 (Cl:A3-1; AbD Serotec, Raleigh, NC), anti-MCP-1 (ab8101; Abcam), and anti-Ly5.1 and anti-Ly5.2 (110718, 109816; Biolegend, San Diego, CA). The sections were examined under a microscope equipped with a digital camera (Olympus Co., Tokyo, Japan) or a BZ-H1M analyzing system (Keyence, Osaka, Japan). The immunofluorescence intensity of F4/80 was calculated by summing the fluorescent areas using ImageJ software (National Institutes of Health, Bethesda, MD).

##### CNV Quantification

One week after laser treatment, the mice were euthanized, and choroidal flat mounts were prepared and incubated with FITC-conjugated isolectin (Invitrogen) to visualize the CNV. The images were obtained at 1-μm intervals from the surface to the deepest focal plane using an FV 1000 confocal fluorescence microscope (Olympus). The CNV volume was calculated by summing the fluorescent areas using ImageJ software as described previously (33).

##### Bone Marrow Transplantation

Mouse bone marrow transplantation (BMT) was performed as previously described (26). In brief, *Angptl2* KO and WT recipient mice underwent 9 grays total body irradiation to eradicate bone marrow cells (BMCs), and then BMCs from *Angptl2* KO or WT mice were transplanted intravenously. Six weeks after transplantation, the replacement of more than 90% of peripheral blood cells in the recipient mice with donor cells was confirmed by detecting Ly5.1 and Ly5.2 in the peritoneal blood of the Ly5.1-WT BMC transplanted Ly5.2-WT hosts using flow cytometry. Seven weeks after transplantation, the recipient mice were anesthetized, followed by laser photocoagulation to induce CNV.

##### Peritoneal Macrophages

Peritoneal macrophages were obtained by injecting 3 ml of 4% brewer's thioglycollate (Merck) intraperitoneally followed by collecting the elicited peritoneal exudate cells 4 days after injection. Exudate cells were centrifuged and resuspended in RPMI medium (Life Technologies) with 100 units/ml of penicillin and 100 μg/ml of streptomycin (Nacalai Tesque, Kyoto, Japan) and 10% FBS (Lonza, Walkersville, MD) at 37 °C with 5% CO_2_ for 24 h.

##### RAW264.7 Cell Line

RAW264.7 cells were maintained in DMEM (Sigma-Aldrich) supplemented with 100 units/m of penicillin and 100 μg/m of streptomycin (Nacalai Tesque) and 10% FBS (Lonza). The cells were incubated at 37 °C and 5% CO_2_ in a humidified atmosphere. The culture medium was replaced three times each week.

##### Cell Treatments

The peritoneal macrophages or RAW264.7cells were cultured with serum-free medium for 24 h and then stimulated with 10 μg/ml recombinant Angptl2 (IBL, Fujioka, Japan). For treatment with neutralizing antibodies or inhibitors, the cells were pretreated with 10 μg/ml neutralizing antibodies, integrin α4 antibody (BD Biosciences, San Jose, CA), integrin β2 antibody (BD Biosciences), and integrin α5β1 antibody (Merck Millipore), or control IgG (Calbiochem, San Diego, CA) 30 min before stimulation of recombinant Angptl2 (IBL) or vehicle. Alternatively, the cells were pretreated with either 1 μg/ml DHMEQ (Provided by Dr. Umezawa), 10 μm U0126 (Promega, Tokyo, Japan), or Vehicle (0.1% DMSO) 30 min before stimulation of Angptl2 or vehicle.

##### Real Time RT-PCR

Total RNA of the RPE-choroid, peritoneal macrophages with or without stimulation, and RAW264.7 cells stimulated for 3 h by Angptl2 or vehicle was extracted with TRIzol reagent (Invitrogen), and cDNA was prepared using the SuperScript VILO Master Mix (Invitrogen) according to the manufacturer's instructions. Real time PCR was performed with the SYBR Green PCR Master Mix Kit (Applied Biosystems, Austin, TX), and the mRNA levels were normalized to *Gapdh* levels. Gene-specific primers are as follows: *Angptl2* forward, 5′-GGA GGT TGG ACT GTC ATC CAG AG-3′; *Angptl2* reverse, 5′-GCC TTG GTT CGT CAG CCA GTA-3′; *Il-1*β forward, 5′-TCC AGG ATG AGG ACA TGA GCA C-3′; *Il-1*β reverse, 5′-GAA CGT CAC ACC AGC AGG TTA-3′; *Il-6* forward, 5′-AAG TCG GAG GCT TAA TTA CAC ATG T-3′; *Il-6* reverse, 5′-CCA TTG CAC AAC TCT TTT CTC ATT C-3′; *Mmp-9* forward, 5′-GCC CTG GAA CTC ACA CGA CA-3′; *Mmp-9* reverse, 5′-TTG GAA ACT CAC ACG CCA GAA G-3′; *Tgf*-β*1* forward, 5′-TGG AGC AAC ATG TGG AAC TC-3′; *Tgf*-β*1* reverse, 5′-CGT CAA AAG ACA GCC ACT CA-3′; *F4/80* forward, 5′-GAG ATT GTG GAA GCA TCC GAG AC-3′; *F4/80* reverse, 5′-GAT GAC TGT ACC CAC ATG GCT GA-3′; integrin α4 forward, 5′-CAG AGC CAC ACC CAA AAG TTA-3′; integrin α4 reverse, 5′-GGT GAA ATG TCG TTT GGG TC-3′; integrin α5 forward, 5′-AGG AGT TCC AAG AGC AA-3′; integrin α5 reverse, 5′-ATC CAA AAT ACG CAG CCA TC-3′; integrin β1 forward, 5′-TGG AAA ATT CTG CGA GTG TG-3′; integrin β1 reverse, 5′-GCA TTC ACA AAC ACG ACA CC-3′; integrin β2 forward, 5′-GTA CAG GCG CTT TGA GAA GG-3′; integrin β2 reverse, 5′-TTT CAG CAA ACT TGG GGT TC-3′; *Cd4* forward, 5′-AAG CGA GAC CTG GGG TAT CT-3′; *Cd4* reverse, 5′-TCC TTC CCA CTC AAC TTT GC-3′; *Cd8a* forward, 5′-GCC GAC AAT CTT CTG GTC TC-3′; *Cd8a* reverse, 5′-TCA GTT CTG TCG TGC CAG TC-3′; *Cd138* forward, 5′-GCT CTG GGG ATG ACT CTG AC-3′; *Cd138* reverse, 5′-AAA GCA GTC TCG GTG TTG CT-3′; *Mcp-1* forward, 5′-GCA TCC ACG TGT TGG CTC A-3′; *Mcp-1* reverse, 5′-CTC CAG CCT ACT CAT TGG GAT CA-3′; *RelA* forward, 5′-GTA TTC CTG GCG AGA GAA GC-3′; *RelA* reverse, 5′-CTT GGT GAC CAG GGA GAT TC-3′; *Mapk1* forward, 5′-CAC CAA CCT CTC GTA CAT CG-3′; *Mapk1* reverse, 5′-GAC TGA TTT TCT TGA TAG CAA CTC G-3′; *Mapk3* forward, 5′-ACA CGC AGC TGC AGT ACA TC-3′; *Mapk3* reverse, 5′-CAA AGG GGC TGA TCT TG-3′; *Gapdh* forward, 5′-AGG AGC GAG ACC CCA CTA AC-3′; and *Gapdh* reverse, 5′-GAT GAC CCT TTT GGC TCC AC-3′.

##### Immunoblot Analysis

Lysates of cultured peritoneal macrophages and RAW264.7 cells were prepared 1 h after Angptl2 or vehicle stimulation following each treatment except for the analysis of phosphorylated ERK in the peritoneal macrophages, which were prepared 30 min after the stimulation according to each peaking time point. The lysates were electrophoretically separated and electrically transferred to a PVDF membrane (Immobilon-P; Merck Millipore). The membrane was incubated with TNB blocking buffer (0.1 m Tris-HCl, pH 7.5, 0.15 m NaCl, 0.5% TSA blocking reagent; PerkinElmer Life Sciences) and then incubated with anti-phospho-NFκB p65, anti-NFκB p65, and anti-phospho-ERK1/2 (all from Cell Signaling Technology, Beverly, MA), and anti-Gapdh (Sigma) followed by incubation with goat HRP anti-rabbit or mouse IgG (Jackson ImmunoRes, West Grove, PA) as secondary antibodies. Signals were detected using an ECL system (Amersham Biosciences ECL Western blotting System; GE Healthcare Life Science), measured using ImageJ software, and normalized to GAPDH.

##### Knockdown Analyses

Knockdown analyses were performed according to the manufacturer's protocols. Briefly, 20 pmol of either *RelA* or *Mapk1* together with *Mapk3* siRNAs (Stealth RNAi, Life Technologies) were diluted into 100 μl of OPTI-MEM with 1 μl of Lipofectamine® RNAi MAX (Life Technologies) for 20 min. Then this solution was added to RAW 264.7 cells that had been trypsinized and suspended in 500 μl of DMEM containing 10% FBS at a concentration of 1 × 10^6^ cells/ml for transfection. The cells were utilized 24 h after the incubation.

##### Cell Migration Assays

Cell migration assays were performed using the Cultrex® Cell Invasion Assay (96-well, 8-μm pore, 3457-096-K; Trevigen Inc., Gaithersburg, MD). Cells (5 × 10^5^) were added to the upper reservoir either with 1 μg/ml DHMEQ, 10 μm U0126, or vehicle (0.1% DMSO). DMEM-12 with or without 10 μg/ml recombinant Angptl2 was placed in the lower reservoir. After 24 h of migration, the assay was performed according to the manufacturer's instructions.

##### MTT Assay

Cells treated with each inhibitor were subjected to MTT analysis using a Cell Proliferation kit (Roche Life Science), according the manufacturer's protocol. Briefly, confluent cells in 24-well dishes stimulated by 1 μg/ml DHMEQ, 10 μm U0126, or Vehicle (0.1% DMSO) were incubated with MTT solution for 3 h and dissolved in DMSO followed by measurement using an absorption spectrophotometer at a wavelength of 570 nm.

##### Statistical Analysis

All results are expressed as the means ± S.D. Differences were analyzed using the unpaired *t* test when comparing two groups and the one-way analysis of variance with Tukey's post hoc test when comparing three or more groups, and they were considered significant at *p* < 0.05.

## Results

### 

#### 

##### Angptl2 Induction and Macrophage Recruitment Precede CNV Development

**FIGURE 1.**
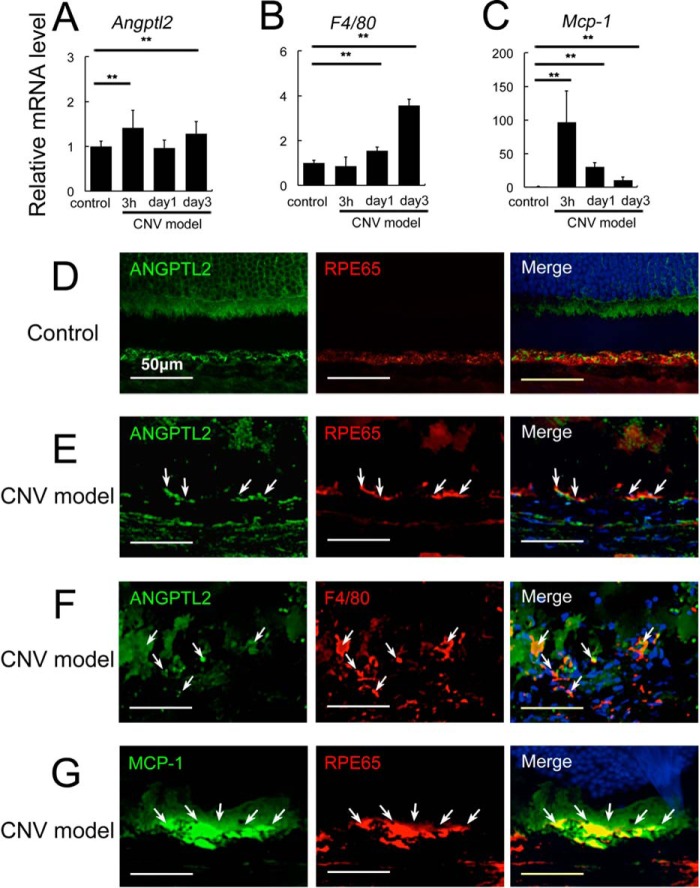
**Angptl2 expression and macrophage recruitment during CNV generation.** *A–C*, cytokines and macrophage induction after laser treatment to induce CNV in the RPE-choroid, determined by real time RT-PCR. *A* and *B*, the mRNA levels of *Angptl2* (*A*) were increased in a biphasic manner, both rapidly and at 3 days after the laser treatment for CNV induction, although up-regulation of *F4/80* (*B*), a macrophage marker, began at 1 day and was further elevated at 3 days. *C*, the mRNA levels of *Mcp-1* rapidly peaked and were still increasing until 3 days compared with the control. *D–G*, immunostaining with or without laser treatment. *D* and *E*, Angptl2 (*green*) and an RPE marker, RPE65 (*red*), were co-expressed under control conditions (*D*) and at 3 h after CNV induction (*E*). *F* and *G*, Angptl2 (*green*) and a macrophage marker, F4/80 (*red*), were also co-expressed 3 days after CNV induction (*F*), and MCP-1 (*green*) and RPE65 (*red*) were co-expressed 3 h after CNV induction (*G*). *Arrows* in each panel indicate sites of co-expression. **, *p* < 0.01. *Scale bar*, 50 μm.

First, we checked whether Angptl2 was induced in the laser-induced CNV model that is used extensively in animal studies of wet AMD (30). The *Angptl2* mRNA levels were significantly up-regulated in the RPE-choroid complex sample in a biphasic manner; as early as 3 h and 3 days after the local laser treatment used to induce CNV (Fig. 1*A*). Macrophage marker *F4/80* mRNA was already induced at 1 day and further elevated at 3 days, consistent with previous report (34) (Fig. 1*B*). The mRNA levels of *Mcp-1*, which is released from the RPE and induces macrophage recruitment (8, 35), were also up-regulated shortly after the laser treatment and increased until day 3 (Fig. 1*C*).

We further examined which cells express Angptl2 in the CNV model by co-immunostaining for Angptl2 with the RPE marker, RPE65, or a macrophage marker, F4/80. Angptl2 was clearly expressed in the RPE under control conditions (Fig. 1*D*), at 3 h (Fig. 1*E*) and 3 days (data not shown) after CNV induction and also in the macrophages 3 days after CNV induction (Fig. 1*F*) when the macrophages had been substantially recruited (Fig. 1*B*). These results indicated that the Angptl2 cytokine can be secreted from both RPE and macrophages during the process of CNV induction. We also confirmed that MCP-1 was expressed in the RPE as early as 3 h after CNV induction (Fig. 1*G*). These findings suggested that Angptl2 and MCP-1 induced by the laser treatment for CNV development might have contributed to macrophage recruitment to the focal area and that the recruited macrophages could have further produced Angptl2 in the RPE-choroid, which might in turn have resulted in persistent recruitment of the macrophages.

##### Angptl2-deficient Mice Exhibit Reduced CNV Volume and Suppressed Levels of Inflammatory Mediators

**FIGURE 2.**
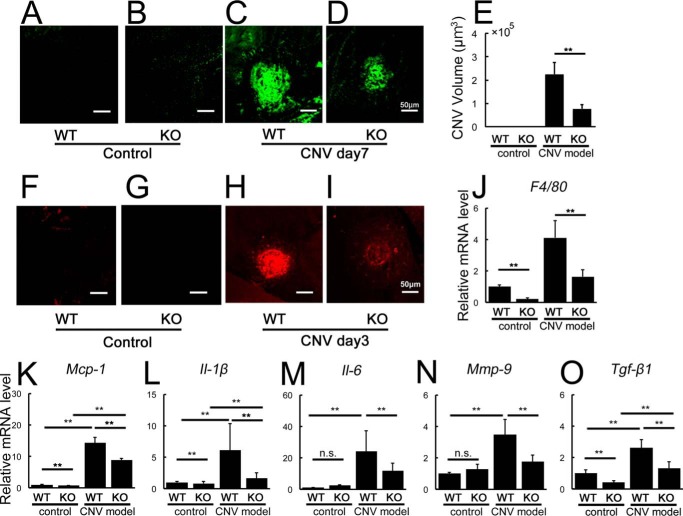
***Angptl2* KO mice exhibit reduced CNV and suppressed levels of inflammatory mediators in the laser-induced CNV model.** *A–E*, volumes of CNV determined with lectin staining 7 days after induction. *A* and *B*, no CNV was observed without laser treatment. *C–E*, laser-induced CNV was suppressed in *Angptl2* KO compared with WT mice. *F–K*, macrophage-related analyses of the RPE-choroid 3 days after laser treatment. Immunostaining with the macrophage marker F4/80 (*F–I*) and mRNA levels of *F4/80* (*J*) and *Mcp-1* (*K*) as measured by real time RT-PCR were suppressed in the KO mice after CNV induction. mRNAs of *F4/80* and *Mcp-1* were already down-regulated in the KO mice at baseline. *L–O*, real time RT-PCR analyses of inflammatory mediators performed 3 days after laser treatment. Laser-induced increases in *Il-1*β (*L*), *Il-6* (*M*), *Mmp-9* (*N*), and *Tgf*-β*1* (*O*) mRNAs in the RPE-choroid were suppressed in the KO mice. *KO*, *Angptl2* knock-out. *n* = 12. **, *p* < 0.01. *Scale bar*, 50 μm.

To address the role of Angptl2 in CNV, we analyzed WT and *Angptl2* KO mice in the CNV model. We found that the volume of induced CNV in *Angptl2* KO mice was significantly reduced compared with that in WT mice at 7 days following laser treatment (Fig. 2, *A–E*). During this process, at 3 days after laser treatment, fewer macrophages were recruited to the developing lesion in *Angptl2* KO mice than in WT mice, as shown by the immunofluorescence staining of F4/80 (Fig. 2, *F–I*) and *F4/80* mRNA levels in the RPE-choroid (Fig. 2*J*). Consistent with these findings, laser-induced increases in the mRNA levels of *Mcp-1* were suppressed in the RPE-choroid of *Angptl2* KO mice *versus* WT controls (Fig. 2*K*), indicating that Angptl2 regulated *Mcp-1* expression. Furthermore, at 3 days after laser treatment, inflammatory mediators including *Il-1*β, *Il-6*, *Mmp-9*, and *Tgf*-β*1* (Fig. 2, *L–O*), all of which accelerate CNV formation, were also attenuated in the RPE-choroid of the *Angptl2* KO mice. These findings indicated that Angptl2 promotes CNV generation, inducing the observed inflammation and macrophage recruitment.

##### Angptl2 Derived from Both Transplanted Bone Marrow and the Host Promotes CNV Development

**FIGURE 3.**
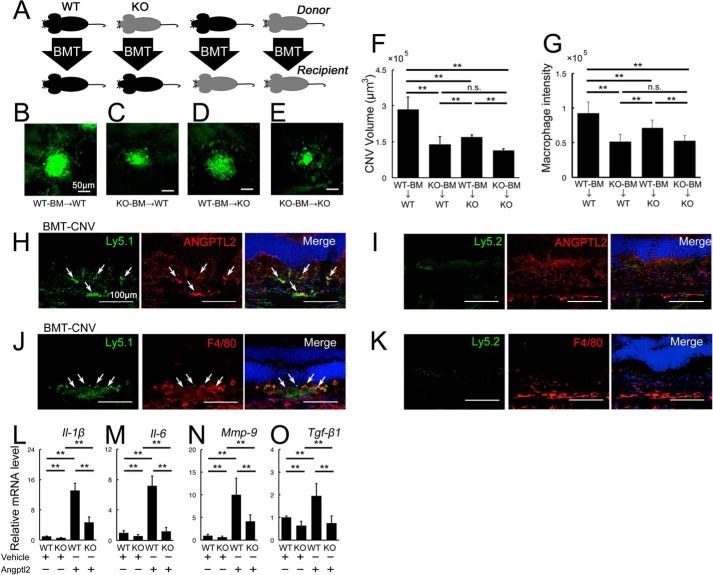
**Angptl2 derived from both transplanted BMCs and the host contribute to laser-induced CNV development.** *A–G*, BM-transplanted mice were exposed to laser photocoagulation, and the CNV volumes were measured 7 days later. *A*, cartoon depicting the various combinations of donor and recipient genotypes. WT is shown in *black*, and KO is in *gray. B–F*, lectin-stained CNV from BMC-transplanted mice 7 days after laser treatment. *Scale bar*, 50 μm. *B* and *C*, compared with WT-BMC-transplanted WT hosts (*B*), KO-BMC-transplanted WT hosts (*C*), and WT-BMC-transplanted KO hosts (*D*) developed CNV with reduced volumes. *E*, the CNV volumes were further reduced in KO-BMC-transplanted KO hosts. *F*, graphical representation of the average CNV volume in each group of BMC-transplanted mice. *G*, immunofluorescence intensity of F4/80, a macrophage marker, in the BM-transplanted mice 3 days after laser treatment. The macrophage level was comparable to the CNV volume in each group. *H–K*, double immunostaining of the retinal sections with the BMC markers, Ly5.1 or Ly5.2, and Angptl2, in the retinal sections of Ly5.1-positive BMC-transplanted Ly5.2-positive hosts in which Ly5.2-positive BMCs were eliminated by x-ray irradiation prior to BMT. Ly5.1-positive transplanted BMCs expressed Angptl2 (*H*, *arrows*) and F4/80 (*J*, *arrows*), but Ly5.2-positive BMCs were not observed in the lesion (*I* and *K*). *L–O*, real time RT-PCR analyses of inflammatory mediators in the peritoneal macrophages harvested from WT and KO mice. All the mRNAs of *Il-1*β (*L*), *Il-6* (*M*), *Mmp-9* (*N*), and *Tgf*-β*1* (*O*) were suppressed in the macrophages of the KO mice, whereas all the mRNAs were up-regulated in response to additional recombinant Angptl2 protein in the macrophages of both WT and KO mice. *KO*, Angptl2 knock-out. *Scale bar*, 50 μm. *n* = 10. **, *p* < 0.01.

We next asked which cells were responsible for Angptl2 production during CNV generation. We performed BMT in which BMCs from *Angptl2* KO or WT mice were transplanted into WT or KO hosts after eradicating the host BMCs by x-ray irradiation (Fig. 3*A*). The laser treatment was performed to induce CNV at 7 weeks from BMT, after engraftment of the donor BMCs was confirmed by flow cytometric analysis of the peripheral blood at 6 weeks from BMT.

The laser-induced CNV volumes (Fig. 3, *B–F*) showed that KO donor BMCs (KO-BMC) transplanted into WT hosts led to a significant reduction in CNV development in comparison with WT-BMC transplantation into WT hosts (Fig. 3, *B*, *C*, and *F*), indicating that BMC-derived Angptl2 played an important role in promoting CNV. The result that the KO-BMC-transplanted KO host showed reduction in the size of CNV compared with the WT-BMC-transplanted KO host was also consistent with this idea (Fig. 3, *D–F*). Comparison of the effect of WT-BMC transplantation into WT and KO hosts showed that CNV development was significantly reduced in the KO hosts (Fig. 3, *B*, *D*, and *F*), suggesting that Angptl2 produced in the focal tissues of the host also contributed to CNV formation in this model. On the other hand, there were no differences in CNV development in WT or KO hosts transplanted with KO-BMCs (Fig. 3, *C*, *E*, and *F*).

Furthermore, macrophage recruitment was parallel to the CNV volume; compared with the WT-BMC transplanted WT hosts, fewer macrophages were recruited in the WT-BMC transplanted KO hosts and in the KO-BMC transplanted WT hosts (Fig. 2*G*). There were also no differences in macrophage recruitment in WT or KO hosts transplanted with KO-BMCs.

To confirm that the BM-derived macrophages were recruited to the local lesion and expressed Angptl2 during CNV development, we transplanted Ly5.1-positive Angptl2-positive BMCs into Ly5.2-positive Angptl2-positive mice in which x-ray irradiation had been performed before BMT to eliminate Ly5.2-positive BMCs and analyzed the resulting retinal sections (Fig. 3, *H–K*). Ly5.1 and Ly5.2 represent leukocyte common antigens and are markers for BMCs. The Ly5.1-positive cells were recruited to express Angptl2 in the local lesion 3 days after CNV induction, indicating that the BM-derived cells were recruited and expressed Angptl2 during CNV development (Fig. 3*H*). In addition, the Ly5.1-positive cells were positive for F4/80, indicating that the BM-derived cells recruited to the lesion were macrophages (Fig. 3*J*). As expected, no Ly5.2-positive cells were recruited to the lesion (Fig. 3, *I* and *K*).

Taken together, these results indicate that Angptl2 derived from both the BMCs and the host focal cells contributed to macrophage recruitment and the resulting CNV development. In addition, the BMC-derived Angptl2 might play a dominant role in promoting CNV development because no significant host effect on CNV was observed when KO-BMCs were transplanted into WT or KO hosts.

##### Angptl2 Is Required for Induction of Inflammatory Mediators in Macrophages

The next question was whether there were differences in the macrophages derived from KO mice. We measured the mRNA levels of *Il-1*β, *Il-6*, *Mmp-9*, and *Tgf*-β*1* in the peritoneal macrophages harvested from the WT and KO mice. Notably, the mRNAs of all these inflammatory mediators were reduced in the KO mice-derived macrophages (Fig. 3, *L–O*). However, when the recombinant Angptl2 was added to the peritoneal macrophages, all the molecules were up-regulated in response to the exogenous Angptl2 protein not only in WT, but in KO mice-derived macrophages as well, indicating that macrophages with or without *Angptl2* deficiency can respond to exogenous Angptl2 (Fig. 3, *L–O*). Taken together, these results suggested that the Angptl2 produced by both macrophages and hosts induced inflammatory mediators in the macrophages in not only an autocrine but also a paracrine manner.

##### Angptl2 Induction of Inflammatory Mediators in Macrophages Is Mediated by Integrins α4 and β2

**FIGURE 4.**
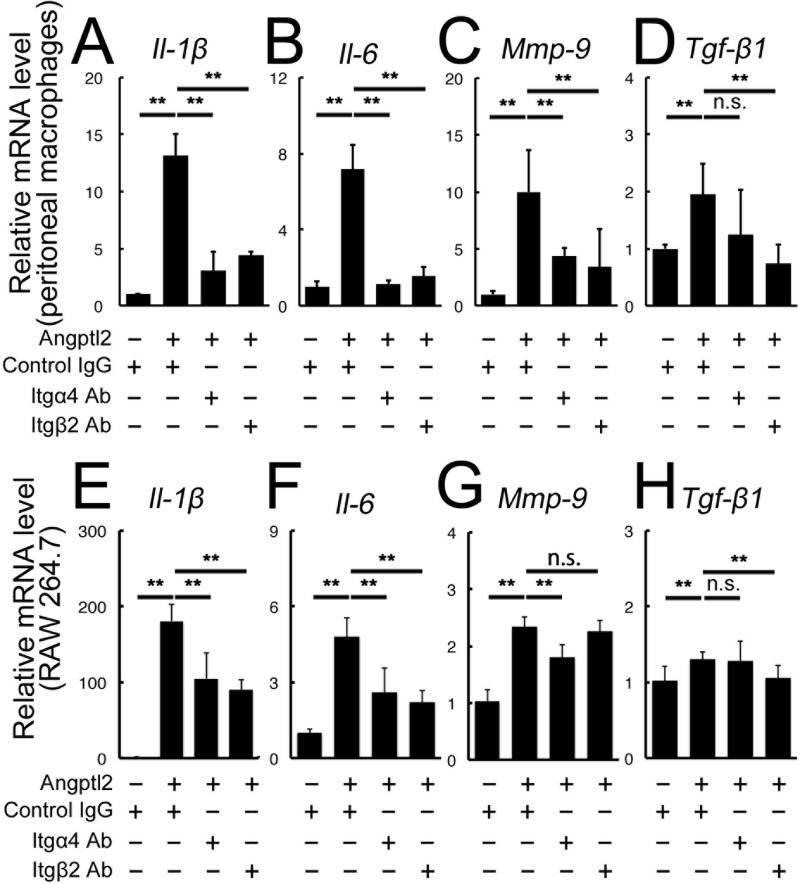
**Angptl2-induced up-regulation of inflammatory mediators in macrophages is mediated by the integrins α4 and β2.** *A–H*, real time RT-PCR analyses of the inflammatory mediators *Il-1*β, *Il-6*, *Mmp-9*, and *Tgf*-β*1* after Angptl2 treatment in the WT-derived peritoneal macrophages (*A–D*) and RAW264.7 cells (*E–H*). All the mRNAs were up-regulated compared with the control culture. *A–D*, in the peritoneal macrophages, treatment with anti-integrin α4 or anti-integrin β2 neutralizing antibodies repressed the Angptl2-induced up-regulation of *Il-1*β (*A*), *Il-6* (*B*), and *Mmp-9* (*C*), and treatment of anti-integrin β2 neutralizing antibody repressed *Tgf*-β*1* (*D*). *E–H*, in the RAW264.7 cells, treatment with anti-integrin α4 or anti-integrin β2 neutralizing antibodies repressed the up-regulation of *Il-1*β (*E*) and *Il-6* (*F*), treatment with anti-integrin α4 antibody repressed *Mmp-9* up-regulation (*G*) and treatment with anti-integrin β2 antibody repressed *Tgf*-β*1* up-regulation (*H*). *n* = 6. **, *p* < 0.01.

Next, we investigated through which pathway Angptl2 can promote inflammatory mediator expression in the macrophages (7, 36, 37). Knowing that integrins function as one of the receptors of Angptl2 (23) and that integrins α4, α5, β1, and β2 at least were up-regulated in the RPE-choroid after laser treatment for inducing CNV (data not shown), we next applied neutralizing antibodies against the integrins in addition to Angptl2 to the WT-derived peritoneal macrophages (Fig. 4, *A–D*). Notably, the presence of anti-integrin α4 or β2 neutralizing antibodies suppressed the mRNAs of *Il-1*β, *Il-6*, and *Mmp-9*, and anti-integrin β2 suppressed *Tgf*-β*1*.

We further incubated cells from the mouse macrophage cell line RAW264.7 with Angptl2 and found that *Il-1*β, *Il-6*, *Mmp-9*, and *Tgf*-β*1* mRNAs were all significantly up-regulated (Fig. 4, *E–H*). In the presence of Angptl2, the induction of *Il-1*β and *Il-6* mRNAs was suppressed by integrin α4 or β2 neutralization; *Mmp-9* mRNA was suppressed by integrin α4 neutralization, and *Tgf*-β*1* mRNA was suppressed by integrin β2 neutralization. In contrast, anti-integrin α1β5 antibody did not suppress these inflammatory mediators except for *Il-1*β (data not shown). These results suggested that Angptl2, produced by the BM-derived and the host RPE during CNV development, induces the production of inflammatory mediators in macrophages that can accelerate CNV development, at least in part, through integrin α4 and/or β2 activation.

##### Angptl2/Integrin Axis-induced Inflammatory Mediators Are Mediated by Nuclear Factor-κB (NF-κB) and ERK Activation

**FIGURE 5.**
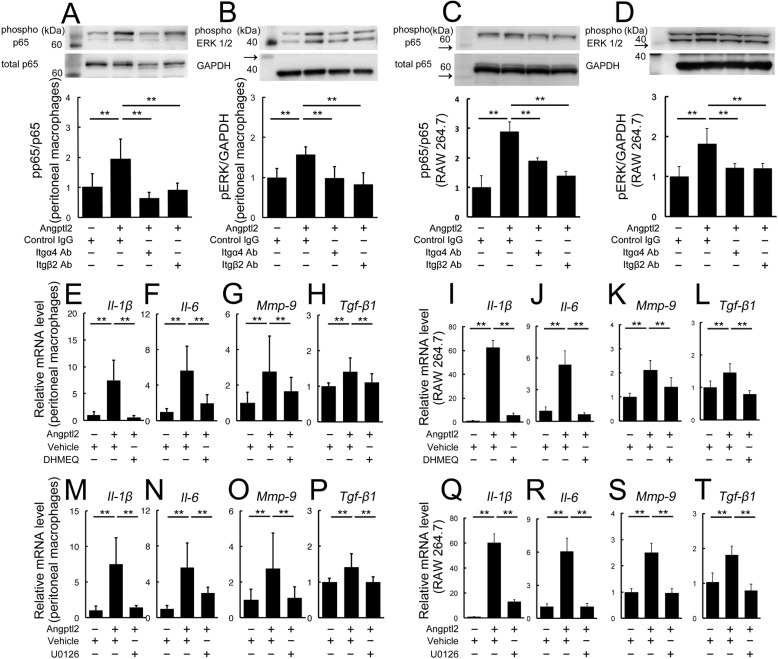
**Angptl2/integrin axis-induced up-regulation of inflammatory mediators is mediated by NF-κB and ERK.** *A–D*, immunoblot analyses for phospho-p65 and phospho-ERK1/2. Angptl2 treatment of WT-derived peritoneal macrophages (*A* and *B*) and RAW264.7 cells (*C* and *D*) induced the phosphorylation and activation of p65 (a component of NF-κB) and ERK1/2, and these activations were suppressed by the presence of anti-integrin α4 and anti-integrin β2 neutralizing antibodies. *Arrows* indicate protein markers. *E–T*, real time RT-PCR analysis of the inflammatory mediator mRNAs *Il-1*β, *Il-6*, *Mmp-9*, and *Tgf*-β*1* in the absence and presence of NF-κB and ERK inhibitors, after stimulation by Angptl2. The up-regulation of all mRNAs by Angptl2 was suppressed by the NF-κB inhibitor DHMEQ (*E–L*) and by the ERK kinase inhibitor U0126 (*M–T*). *n* = 6. **, *p* < 0.01.

We then investigated the intracellular signaling pathways utilized in the Angptl2/integrin axis-mediated production of the inflammatory mediators. Treatment of WT-derived peritoneal macrophages and RAW264.7 cells with Angptl2 resulted in the activation of both NF-κB and ERK1/2, and these activations were inhibited by neutralizing antibodies targeting integrin α4 or β2 (Fig. 5, *A–D*). In addition, mRNAs of all of the Angptl2-induced inflammatory mediators (*Il-1*β, *Il-6*, *Mmp-9*, and *Tgf*-β*1*) were suppressed by incubating the cells with either an NF-κB inhibitor (DHMEQ) (Fig. 5, *E–L*) or an ERK kinase inhibitor (U0126) (Fig. 5, *M–T*) in both WT-derived peritoneal macrophages and RAW264.7 cells, indicating that all these molecules were induced by Angptl2 through both NF-κB and ERK.

**FIGURE 6.**
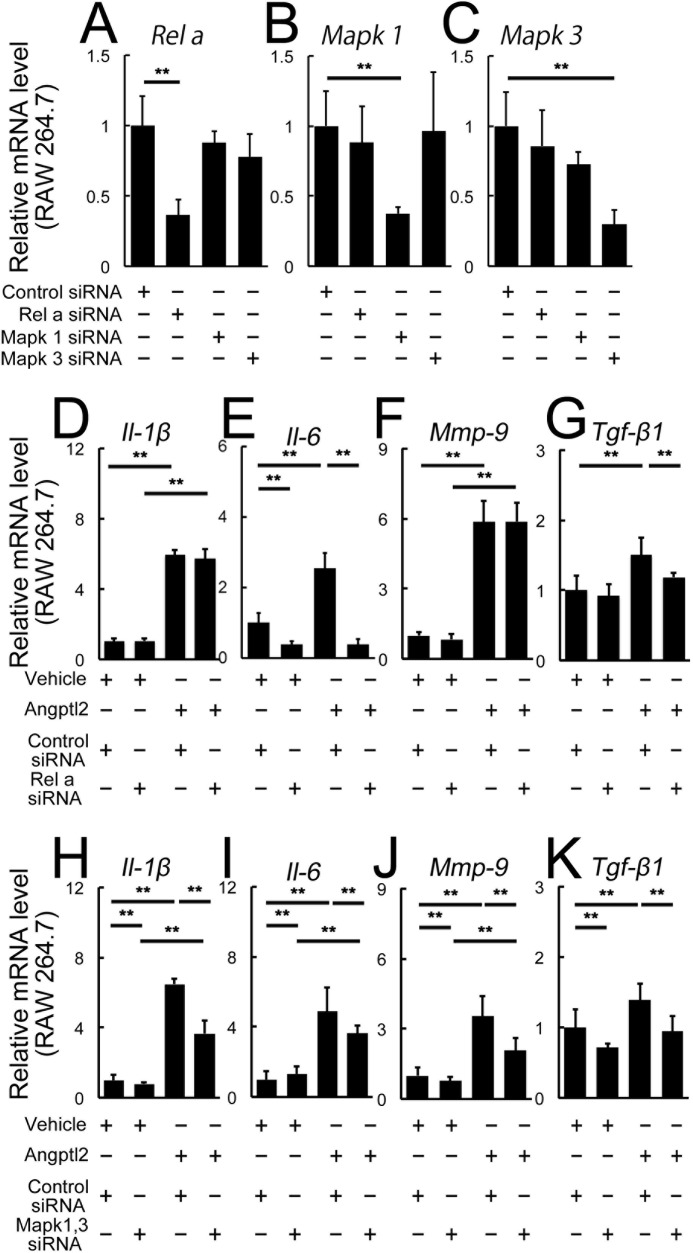
**Roles of RelA (p65) and ERK in Angptl2-induced up-regulation of inflammatory mediators in RAW 246.7 cells.** *A–K*, real time RT-PCR analyses. *A–C*, each siRNA suppressed the levels of the target mRNAs in RAW 264.7 cells. *A*, *RelA*; *B*, *Mapk1*; *C*, *Mapk3. C–G*, *RelA* siRNA suppressed *Il-6* (*E*) and *Tgf*-β*1* (*G*) mRNAs, but not *Il-1*β (*D*) and *Mmp-9* (*F*). *H–K*, co-transfection of *Mapk1* and *Mapk3* siRNAs suppressed all four mediators induced by Angptl2. *n* = 6. **, *p* < 0.01.

The downstream pathways of the Angptl2-induced inflammatory mediators were further confirmed by knockdown experiments using siRNAs either of *RelA*, also known as p65 and one of the components of NF-κB, or *Mapk1* and *Mapk3*, also known as ERK1 and ERK2, in the RAW 264.7 cells (Fig. 6, *A–K*). After confirming knockdown of the target molecules in the transfected cell with *RelA* (*p65*) or co-transfected cells with *Mapk1* and *Mapk3* siRNAs (Fig. 6, *A–C*), the mRNAs of the inflammatory mediators induced by Angptl2 were analyzed (Fig. 6, *D–K*). We found that *RelA* siRNA suppressed *Il-6* and *Tgf*-β*1* mRNAs, although *Il-1*β *and Mmp-9* mRNAs were not affected (Fig. 6, *D–G*), whereas *Mapk1* and *Mapk3* siRNAs suppressed all four mediators induced by Angptl2 (Fig. 6, *H–K*). The discrepancy of the results of *Il-1*β and *Mmp-9* mRNAs obtained by DHMEQ and *RelA* siRNA was most likely because the RelA pathway might not have been involved in inducing these mediators, but other components of NF-κB such as p50, Rel-B, and c-Rel, which are all inhibited by DHMEQ (38), might have been responsible for the NF-κB activation in this context. These findings indicated that the Angptl2/integrin axis-induced production of the inflammatory mediators in macrophages is mediated by the NF-κB and ERK signaling pathways.

##### Angptl2-induced Macrophage Migration is Mediated by NF-κB and ERK Activation

**FIGURE 7.**
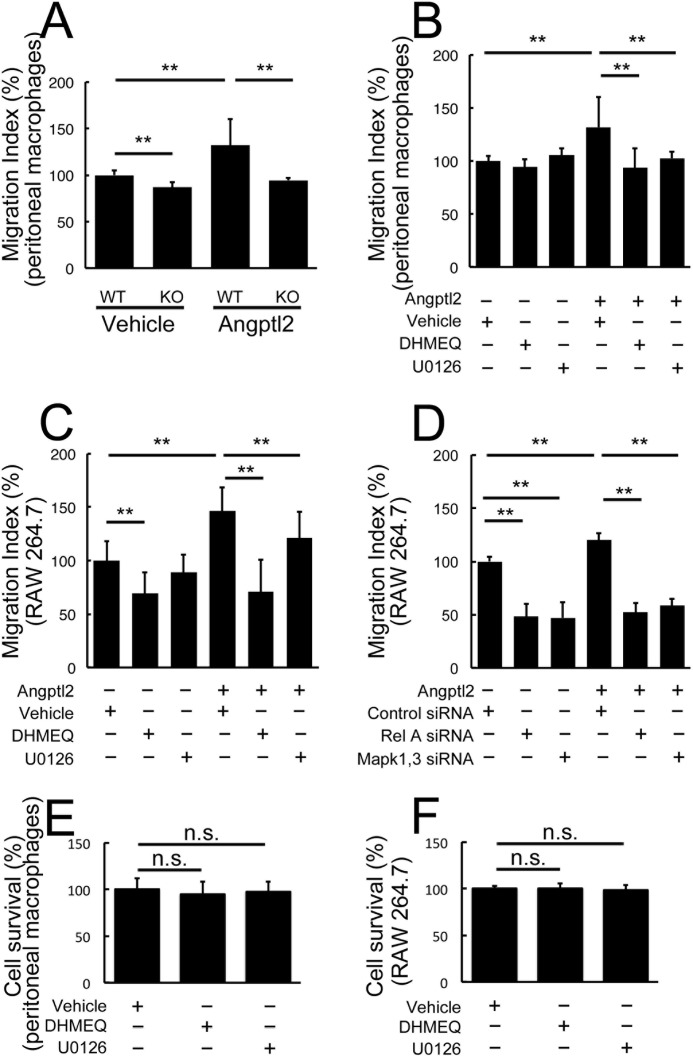
**Angptl2-induced macrophage migration is mediated by NF-κB and ERK activation.** *A–D*, Transwell assays using peritoneal macrophages (*A* and *B*) and RAW264.7 cells (*C* and *D*). Compared with WT-derived macrophages, KO-derived macrophages showed lower migration indices with or without additional Angptl2 (*A*). The migration index was increased by Angptl2 both in the WT-derived peritoneal macrophages (*A* and *B*) and RAW264.7 cells (*C* and *D*). The increase was significantly suppressed either by DHMEQ or U0126 treatment both in the WT-derived peritoneal macrophages (*B*) and RAW cells (*C*). *D*, either transfection of *RelA* siRNA or co-transfection of *Mapk1* and *Mapk3* siRNAs suppressed Angptl2-induced migration in RAW 264.7 cells. *E* and *F*, absence of any changes from either DHMEQ or U0126 treatment in the MTT assay compared with controls is shown. *n* = 8. **, *p* < 0.01.

To evaluate the downstream pathways of Angptl2-induced macrophage recruitment, we performed transmigration assays. First, we showed that KO-derived peritoneal macrophages exhibited a lower migration index compared with WT-derived peritoneal macrophages with or without additional Angptl2 (Fig. 7*A*). After confirming that the migration indices of both WT-derived peritoneal macrophages and RAW264.7 cells were increased in response to Angptl2 (Fig. 7, *A–C*), we analyzed the effects of suppressing NF-κB or ERK using their respective inhibitors, DHMEQ or U0126 (Fig. 7, *B* and *C*). In both cell types, the Angptl2-induced increase in migration was suppressed when cells were treated with either inhibitor (Fig. 7, *B* and *C*). In the RAW264.7 cells, DHMEQ also suppressed migration in the absence of Angptl2, suggesting that the basal level of NF-κB was sufficient to induce migration in this cell line. The migration index was similar to the level observed when DHMEQ was added in the presence of Angptl2. We also confirmed the roles of NF-κB and ERK in the RAW 264.7 cells using siRNAs. As expected, Angptl2-induced migration was suppressed by the addition of siRNA(s) for either *RelA* or *Mapk1* and *Mapk3* (Fig. 7*D*). These findings suggested that Angptl2 also plays a role in macrophage recruitment, which is mediated by NF-κB and ERK activation. The absence of any differences in the MTT assay with or without either inhibitor or siRNAs suggested that these treatments did not affect cell proliferation and survival (Fig. 7, *E* and *F*).

## Discussion

In this study, we demonstrated that Angptl2-deficient mice exhibited suppressed CNV development in a laser photocoagulation model, leading to reduced macrophage infiltration into the lesion and suppressed expression of the inflammatory mediators *Mcp-1*, *Il-1*β, *Il-6*, *Mmp-9*, and *Tgf*-β*1* (Fig. 2). We performed BMT to determine that both BMC- and host-derived Angptl2 contributed to CNV development via recruiting macrophages (Fig. 3). Macrophages required Angptl2 to produce the inflammatory mediators that accelerate CNV development (Figs. 2–4). The inflammatory mediators induced in macrophages were through the binding of Angptl2 to integrin α4 and β2 (Fig. 4), leading to the downstream activation of NF-κB and ERK (Figs. 5 and 6). Angptl2 also induced macrophage migration, which was also mediated by NF-κB and ERK activation (Fig. 7).

The involvement of inflammatory cytokines such as VEGF and MCP-1 in AMD has been extensively studied using laser-induced CNV models (39). In this study, we demonstrated the impact of Angptl2, a pro-inflammatory factor that was previously shown to be associated with the aging process. The tissue expression of Angptl2 increases significantly with aging (15, 24, 25) and is related to obesity (23, 40), rheumatoid arthritis (41), and diabetes (42), disorders that all progress with age. Given that macrophages are involved in AMD and various diseases related to aging and metabolic dysfunction (9, 43), insight into the molecular mechanisms underlying Angptl2-mediated cytokine expression and macrophage migration will contribute to our understanding regarding the pathogenesis of these diseases.

Each of the inflammatory mediators associated with laser-induced CNV is involved in promoting specific steps of CNV development. For example, MCP-1 recruits macrophages, a major step in the development of CNV (7, 35, 37, 44). Our observations that *Angptl2* KO mice exhibited reduced *Mcp-1* expression in the developing lesion and attenuated macrophage recruitment followed by suppressed CNV development were consistent with previous reports. As we showed, MCP-1 was expressed in the RPE preceding macrophage recruitment (Fig. 1). Thus, we postulate that Angptl2 is secreted from local tissues including the RPE and likely from choroid as well, because it can also be expressed in the vascular endothelium (39, 45) in an autocrine and/or paracrine manner, leading to macrophage recruitment.

Other inflammatory mediators were also involved in the CNV development. IL-1β is constitutively expressed in the vascular endothelium and induces vascular sprouting. An IL-1β receptor antagonist suppresses laser-induced CNV development, without reducing macrophage infiltration (11). The contribution of IL-6, a cytokine that acts on both macrophages and the vascular endothelium, to CNV development was shown using *Il-6* KO mice and a neutralizing antibody against the IL-6 receptor (4, 12). MMP-9 is a proteolytic enzyme involved in the breakdown of extracellular matrix. In the eye, it targets type IV collagen in Bruch's membrane (4, 13, 46). MMP-9 activity promotes the infiltration of inflammatory cells into various tissues, and it is reported to mediate the VEGF-induced vascular invasion of tumor cells (47). As expected, *Mmp-9* KO mice exhibit suppressed CNV development in a laser photocoagulation model (13). A previous paper showed TGF-β expression in surgically removed human CNV tissue (48), supporting the involvement of TGF-β in CNV pathogenesis. A recent study showed that TGF-β inhibitor peptides suppress laser-induced CNV, accompanied by a reduction in VEGF levels (14).

**FIGURE 8.**
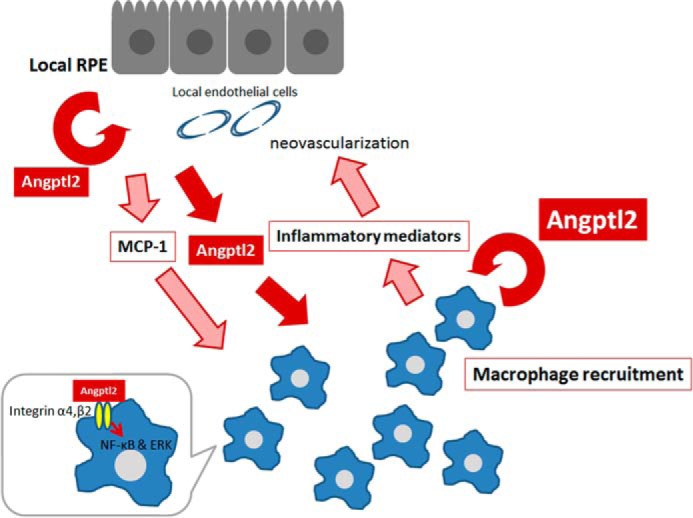
**Proposed mechanism of CNV development related to Angptl2.** Initially, RPE and/or choroid including endothelial cells in the early lesion secrete Angptl2 and MCP-1, both of which in turn recruit macrophages. MCP-1 is at least partly regulated by Angptl2. These macrophages also secrete Angptl2, resulting in the recruitment of additional macrophages. Then Angptl2 derived from both macrophages and RPE and/or choroid induces the expression of inflammatory mediators in macrophages, and these mediators contribute to CNV development. Angptl2 might act in an autocrine and/or paracrine fashion to activate surface-bound integrin α4 and/or β2 and intracellular NF-κB and ERK pathways in macrophages.

Notably, our data showed that the above-described inflammatory mediators were significantly reduced in the RPE/choroid of *Angptl2* KO mice; this was, at least in part, because macrophages required Angptl2 produced by themselves or by the RPE to express these mediators (Figs. 2 and 3). Taken together, these findings suggest that Angptl2 might be a central regulator of multiple inflammatory mediators involved in CNV development. Thus, suppression of Angptl2 might impede multiple steps in the development of CNV (Fig. 8).

Because Angptl2 deficiency in either the host or the transplanted BM-derived cells suppressed CNV (Fig. 3), Angptl2 of both origins might contribute to CNV development. The current results that transplanted BM-derived macrophages were recruited and express Angptl2 (Fig. 3) and that the macrophage marker *F4/80* was up-regulated in the RPE-choroid, whereas other bone marrow-derived inflammatory cell markers such as *Cd4*, *Cd8a*, and *Cd138* were not (data not shown), as well as the fact that macrophages are the main contributor to CNV development (7, 8), indicated that macrophages represent the BMC-derived cells that produce Angptl2 during CNV development. The CNV was significantly reduced by the transfer of Angptl2-deficient BMCs compared with WT BMCs, regardless of whether the host genotype was WT or KO, suggesting that the BMC- or macrophage-derived Angptl2 might have had a greater impact on CNV development than the host Angptl2, possibly because of the higher level of Angptl2 produced by the macrophages.

Although Angptl2 is reported to induce inflammatory mediator production (27, 29, 40, 49), the intracellular pathways involved have been obscure. The results of the current study reveal that Angptl2-induced inflammatory mediator production was mediated at least in part through the activation of surface-bound integrin α4 and/or β2, as well as the downstream activation of NF-κB and ERK.

Previous studies on the involvement of integrins in CNV development were focused on the integrins expressed at the cell surface of the vascular endothelium. The involved subtypes were reported to be α5 and v, and β1 and 3 (50–54). However, considering the substantial contribution of macrophages to CNV (7, 8), our findings that integrins α4 and β2 were involved might be useful in exploring new therapeutic approaches. Notably, the integrins expressed in the aging RPE are reported to be α1–5, αv, and β1 (55). Thus, integrin β2 might represent a novel macrophage target for the treatment of AMD that might not affect normal RPE.

Angptl2-induced macrophage migration was also mediated through the NF-κB and ERK signaling pathways. In a previous report, p38-MAPK was shown to be responsible for osteosarcoma migration and metastasis mediated by Angptl2 (20), suggesting that different signaling pathways might be employed in different cell types. Our data are consistent with the results of a previous study indicating the involvement of NF-κB in Angptl2-mediated macrophage migration (23). However, the role of ERK in this process has not been previously reported.

Based on the data shown here, we propose the following scheme for the involvement of Angptl2 in the pathogenesis of CNV development (Fig. 8). Initially, RPE and/or choroid in the early lesion secretes Angptl2 and MCP-1, both of which in turn recruit macrophages. MCP-1 is also, at least partly, regulated by Angptl2. These macrophages also secrete Angptl2, resulting in the recruitment of additional macrophages. Then Angptl2 derived from both macrophages and RPE and/or choroid induces the expression of inflammatory mediators in macrophages, and these mediators contribute to CNV development. Thus, Angptl2 promotes MCP-1 expression, macrophage recruitment, and the subsequent inflammatory mediator expression. Angptl2 might act in an autocrine and/or paracrine fashion to activate surface-bound integrin α4 and/or β2 and the intracellular NF-κB and ERK pathways in macrophages.

Therefore, focal tissue-derived Angptl2 might trigger CNV, and the macrophage-derived Angptl2 might accelerate the disease pathogenesis, affecting multiple steps. This concept might be applicable to many other Angptl2-related diseases and might explain why Angptl2-related diseases such as chronic kidney disease (56) and type 2 diabetes (42) target different tissues or organs in different patients. It is possible that the variation in local concentrations of Angptl2 in each tissue/organ across individuals determines the lesion sites. Although further studies are required, Angptl2 and its related signaling molecules might be promising targets for new AMD treatments acting on the multiple steps in the pathological pathways.

## Author Contributions

M. H. and Y. Ozawa designed the study and wrote the paper. K. T. performed BMT experiments. H. O. and S. M. performed in vitro experiments including knockdown experiments. E. T. and M. E. performed immunostainings and provided technical assistance. K. U. provided DHMEQ. K. U., K. T., and Y. Oike provided supervision and revised the manuscript, critically. All authors analyzed the results and approved the final version of the manuscript.

## Footnotes

2 The abbreviations used are:

CNV: choroidal neovascularization
AMD: age-related macular degeneration
RPE: retinal pigment epithelium
BMT: bone marrow transplantation
BMC: bone marrow-derived cell
MTT: 3-(4,5-dimethylthiazol-2-yl)-2,5-diphenyltetrazolium bromide
KO-BMC: KO donor BMCs
DHMEQ: dehydroxymethylepoxyquinomicin.
